# Supplementary material for: Microbiome characterization of two fresh pork cuts during production in a pork fabrication facility
Source: Microbiol Spectr. 2025 Jan 30;13(3):e02209-24. doi: 10.1128/spectrum.02209-24 (PMC11878005; doi:10.1128/spectrum.02209-24)
Supplement: Supplemental material — Figure S1 to S8; Table S1. [file spectrum.02209-24-s0006.pdf]

Figure S1 Sequencing depth by Meat Type (BJ = boot jack, TL = tenderloin), before and after QC filtering and decontamination using *decontam*

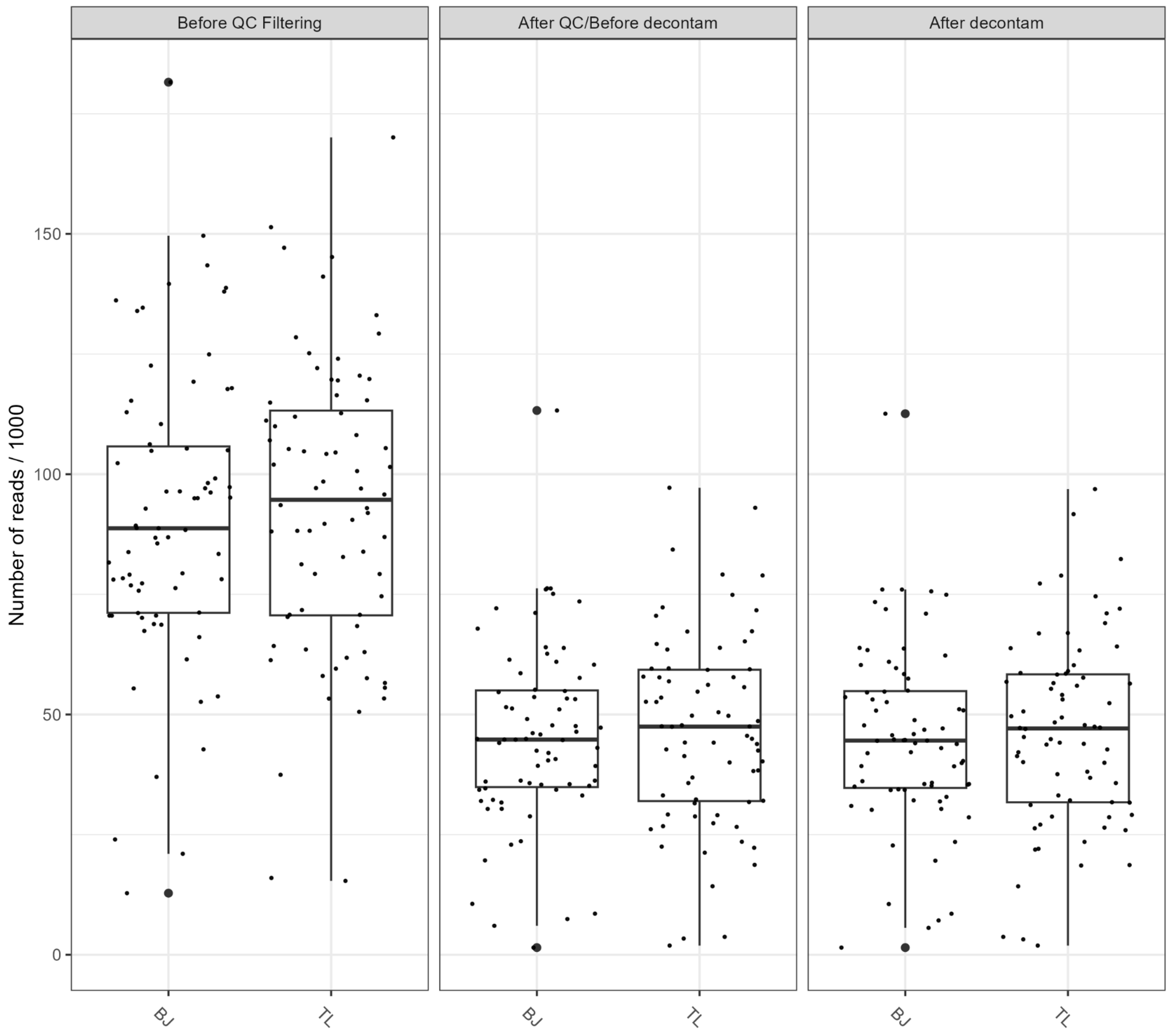

Figure S2 Genus-level relative abundance for the positive control sample processed in this study (“S156”) and the theoretical composition of the Zymo Mock Control (“Mock”)

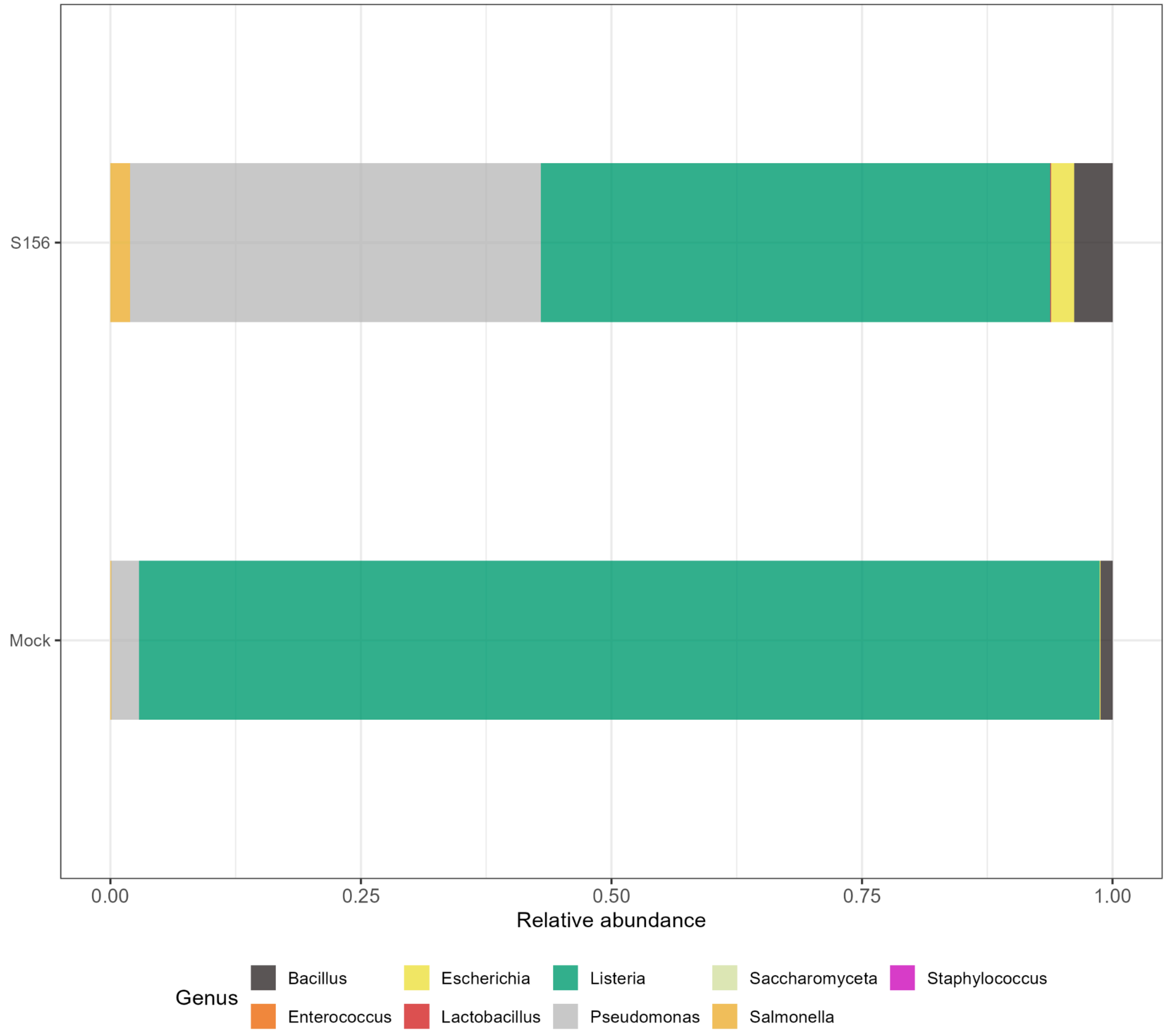

Figure S3 Taxonomic abundance of ASVs removed as contaminants from all sample types (*decontam* package)

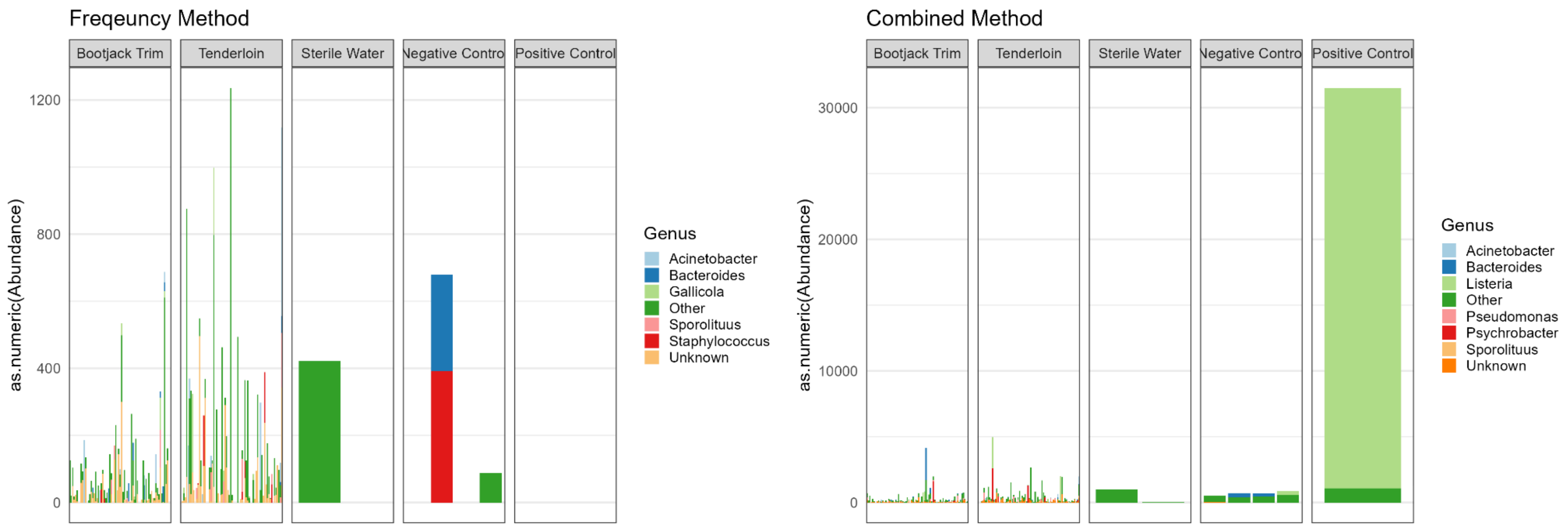

Figure S4 Time series analysis of aerobic plate count (APC) and alpha diversity metrics of Bootjack Trim (yellow) and Tenderloin (gray) by Julian Processing Date. Estimated marginal means were plotted using the geom_smooth function in *ggplot2*, span = 0.5.

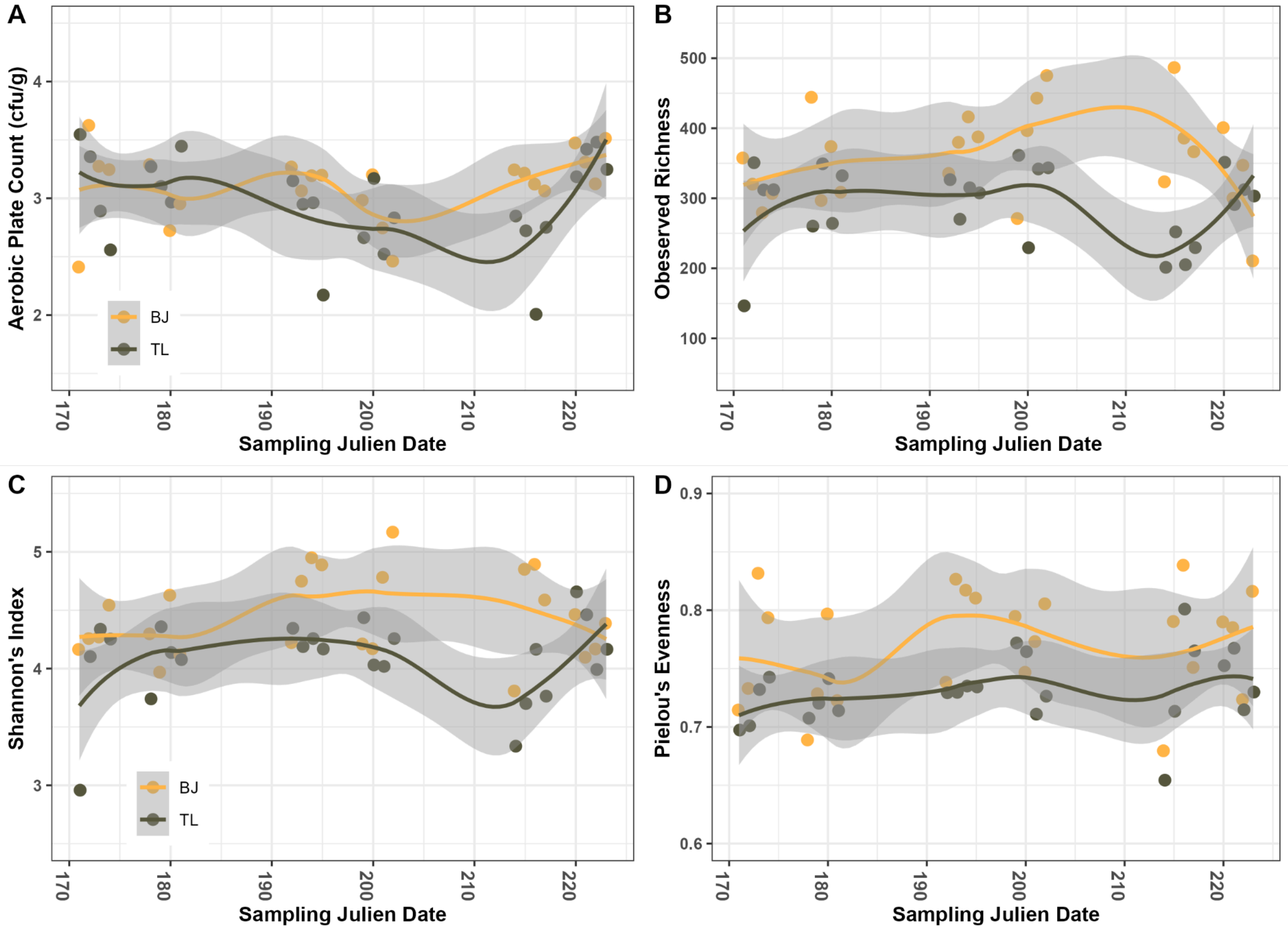

Figure S5 Taxonomic abundance of sequencing reads of individual meat samples at the Phylum level. Taxa with a median less than 100 reads are cumulatively reported as "Other". Each sample is represented by an individual bar on the X-axis. Individual sample information can be found in the supplemental material (SuppS6_Sample.csv).

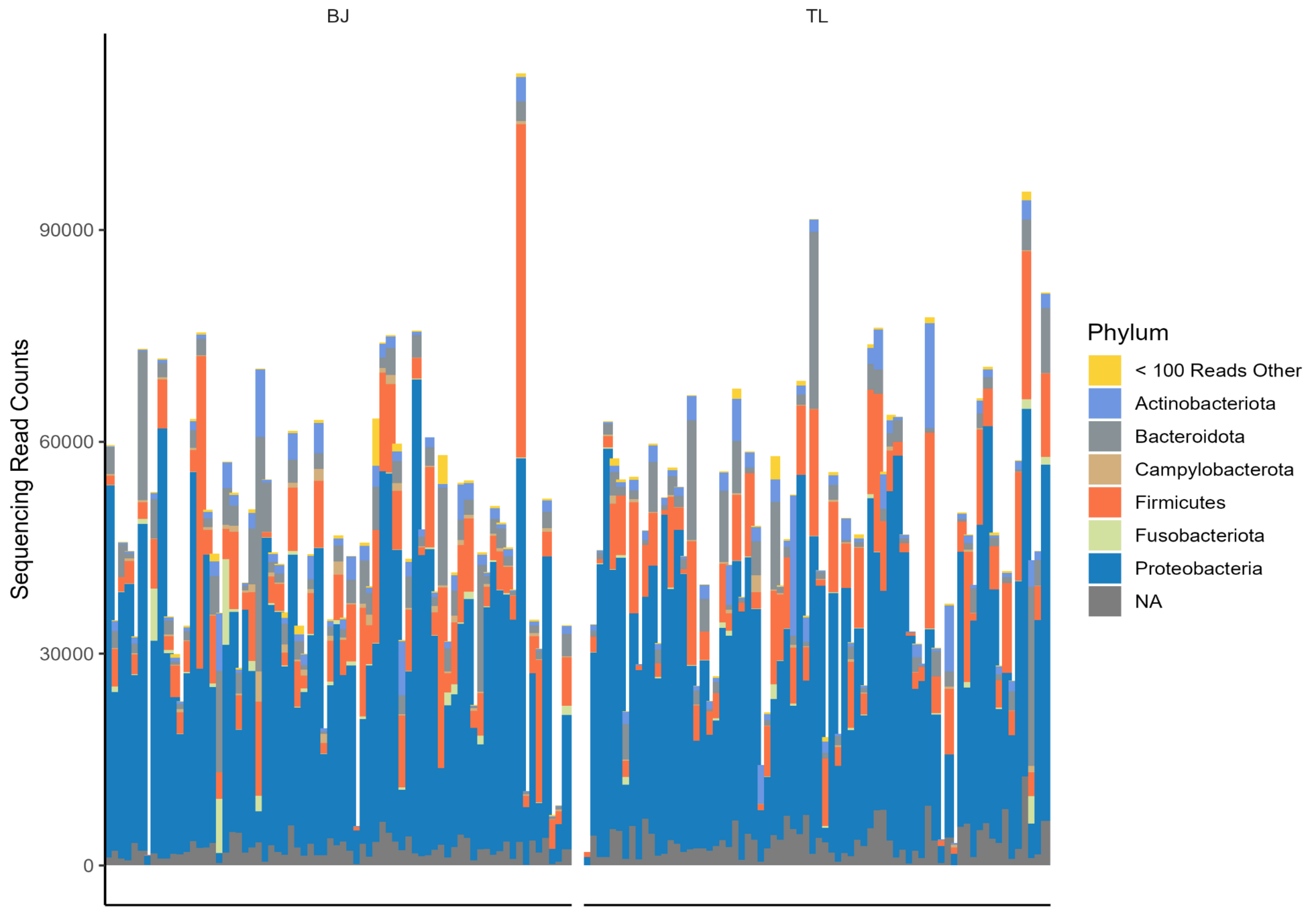

Figure S6 Taxonomic abundance of sequencing reads of individual meat samples at the Genus level. Taxa with a median less than 100 reads are cumulatively reported as “Other”. Each sample is represented by an individual bar on the X-axis. Individual sample information can be found in the supplemental material (SuppS7_Sample.csv).

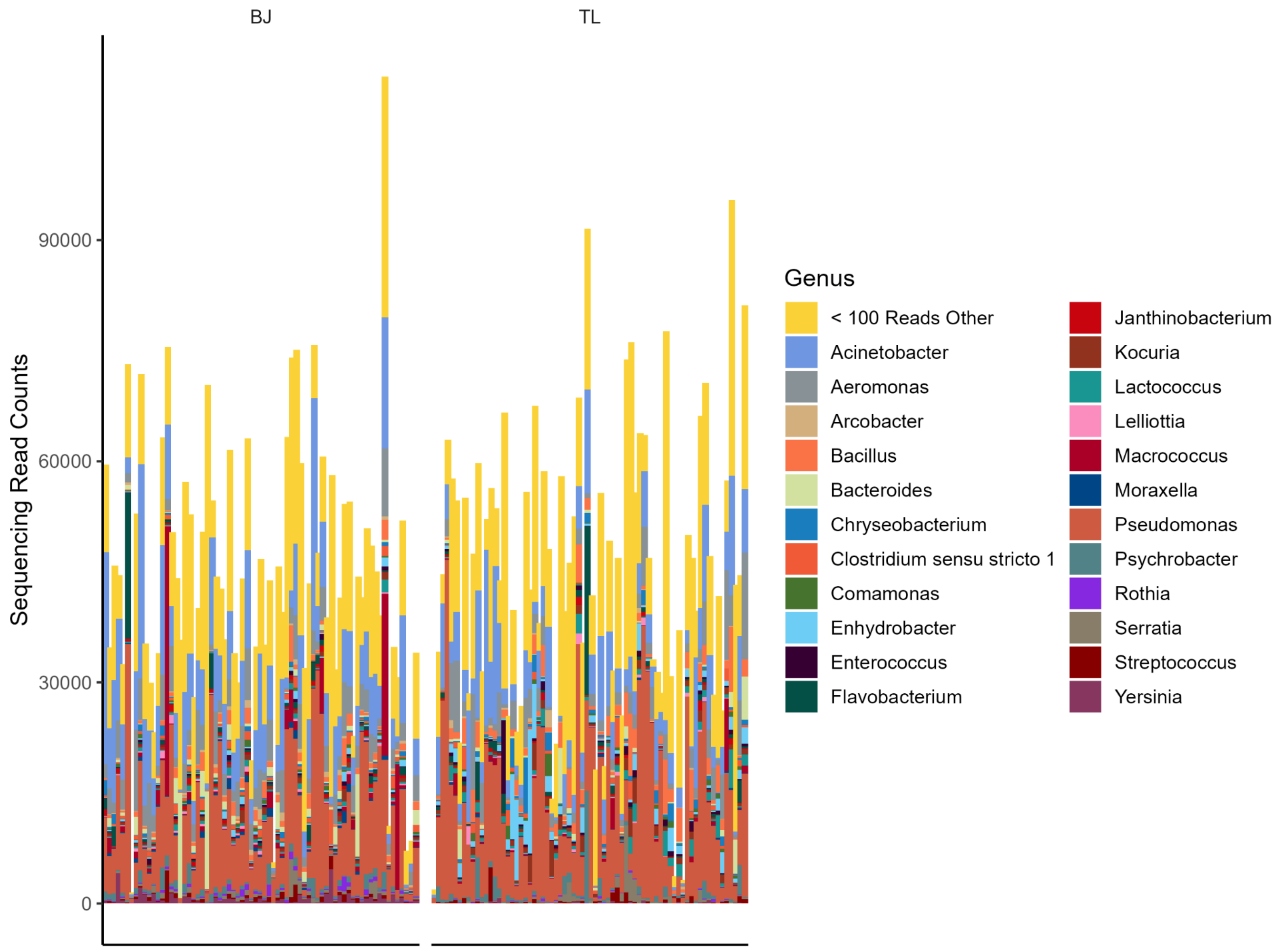

Figure S7 Taxonomic relative abundance of individual meat samples by Meat Type at the Phylum level. Taxa with a median less than 1% relative abundance are cumulatively reported as “Other”. Each sample is represented by an individual bar on the X-axis. Individual sample information can be found in the supplemental material (SuppS8_Sample.csv).

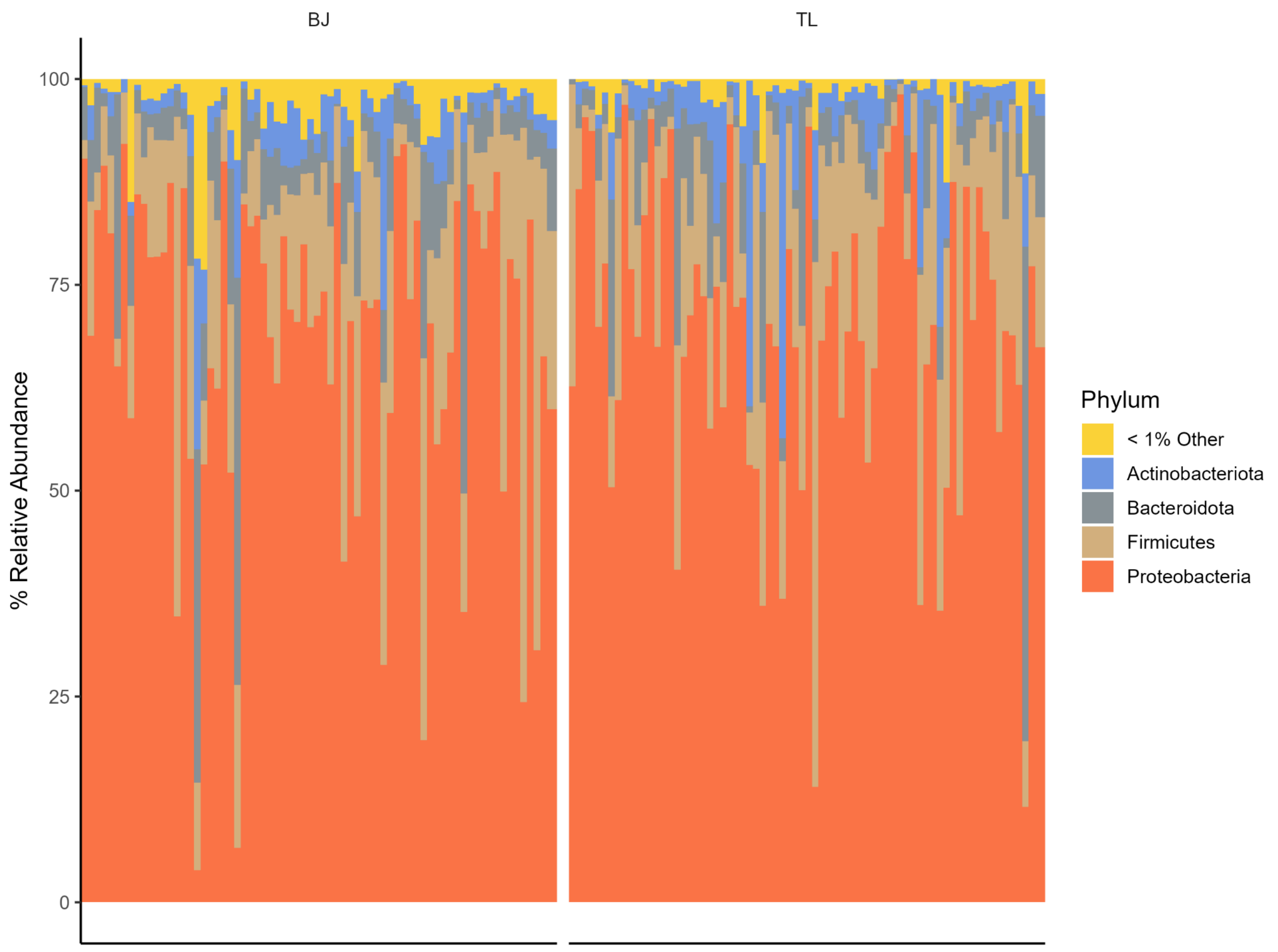

Figure S8 Microbiome diversity of the Bootjack Trim ("BJ", yellow) and Tenderloin ("TL", blue). Estimated marginal means and 95% confidence intervals for (A) aerobic plate count (B) Observed richness (C) Shannon's diversity index (D) Pielou's Evenness.

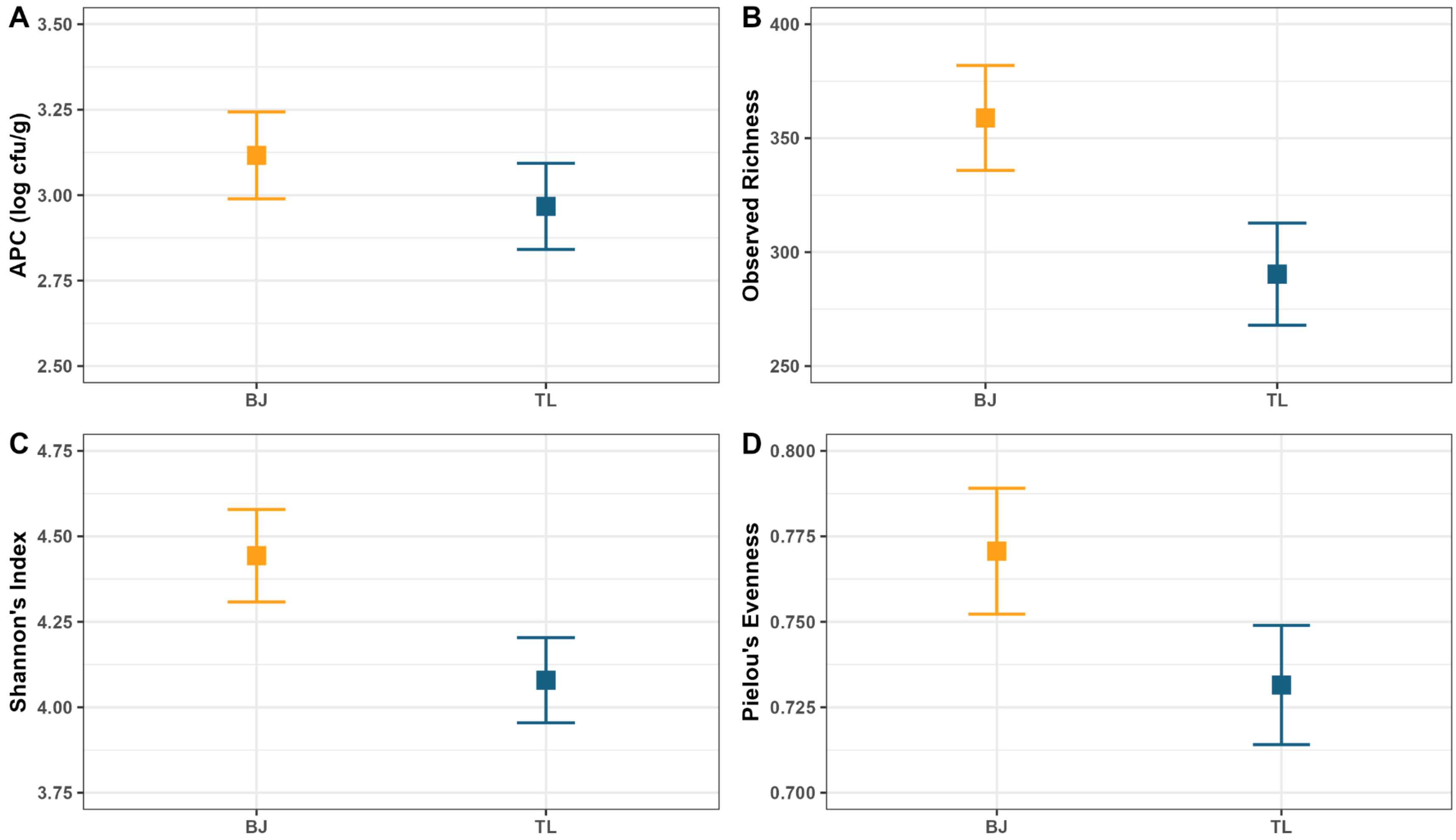

Table S1 Taxa Identified at Each Taxonomic Level By Sequencing Reads and Identified ASV's

Taxonomic_Level	Reads.ID	Percent.Reads.ID	Taxa.ID.ASV.	Percent.Taxa.ID.ASV.
After_ Decontam	6,527,601	100.00%	11,367	100.00%
Kingdom	6,459,818	99.00%	11,040	97.10%
Phylum	6,049,554	92.70%	8,716	76.70%
Order	6,019,119	92.20%	8,314	73.10%
Genus	5,618,906	86.10%	6,117	53.80%
Species	875,367	13.41%	731	6.40%
